# RECIPROCAL RELATIONSHIP BETWEEN LONELINESS AND LIFE SATISFACTION IN HONG KONG OLDER ADULTS

**DOI:** 10.1093/geroni/igae098.3886

**Published:** 2024-12-31

**Authors:** Foong Yee Vivien Tang, Kee Lee Chou

**Affiliations:** The Education University of Hong Kong, Hong Kong, China (People’s Republic); The Education University of Hong Kong, Hong Kong, China (People’s Republic)

## Abstract

Given the limited research on the reciprocal relationship between loneliness and life satisfaction in the Chinese community, this study examines this dynamic and explores the mediating effect of neighbourhood collective efficacy. Loneliness, a prevalent issue among the aging population, significantly impacts psychological well-being and overall quality of life. Conversely, life satisfaction reflects an individual’s overall contentment with life and is a crucial component of successful aging. Utilizing a two-wave secondary data from 1,696 Chinese older adults aged 60 and above (53.9% female), this study analyzed how changes in loneliness predict subsequent changes in life satisfaction, and vice versa. Participants were assessed on their levels of loneliness, life satisfaction, sociodemographic factors, health, social resources, and social support. The findings indicate a reciprocal relationship: loneliness was associated with lower life satisfaction two years later (B = -1.44, p = 0.014), and reduced life satisfaction led to increased loneliness (B = -1.106, p < 0.001). Furthermore, neighbourhood collective efficacy was found to fully mediate the relationship between loneliness and life satisfaction (β = 0.18, p < 0.001). These results underscore the importance of addressing loneliness and life satisfaction simultaneously. Maintaining strong neighbourhood collective efficacy may help mitigate the negative impact of loneliness on life satisfaction. This highlights the potential for targeted, community-based intervention programs designed to enhance life satisfaction and alleviate loneliness among older adults.

